# Prediction and Clinically Important Factors of Acute Kidney Injury Non-recovery

**DOI:** 10.3389/fmed.2021.789874

**Published:** 2022-01-17

**Authors:** Chien-Liang Liu, You-Lin Tain, Yun-Chun Lin, Chien-Ning Hsu

**Affiliations:** ^1^Department of Industrial Engineering and Management, National Yang Min Chiao Tung University, Hsinchu, Taiwan; ^2^Department of Pediatrics, Kaohsiung Chang Gung Memorial Hospital and College of Medicine, Chang Gung University, Kaohsiung, Taiwan; ^3^Department of Pharmacy, Kaohsiung Chang Gung Memorial Hospital, Kaohsiung, Taiwan; ^4^School of Pharmacy, Kaohsiung Medical University, Kaohsiung, Taiwan

**Keywords:** acute kidney injury, kidney function recovery, machine learning, risk prediction, interpretability, electronic health records—EHR

## Abstract

**Objective:**

This study aimed to identify phenotypic clinical features associated with acute kidney injury (AKI) to predict non-recovery from AKI at hospital discharge using electronic health record data.

**Methods:**

Data for hospitalized patients in the AKI Recovery Evaluation Study were derived from a large healthcare delivery system in Taiwan between January 2011 and December 2017. Living patients with AKI non-recovery were used to derive and validate multiple predictive models. In total, 64 candidates variables, such as demographic characteristics, comorbidities, healthcare services utilization, laboratory values, and nephrotoxic medication use, were measured within 1 year before the index admission and during hospitalization for AKI.

**Results:**

Among the top 20 important features in the predictive model, 8 features had a positive effect on AKI non-recovery prediction: AKI during hospitalization, serum creatinine (SCr) level at admission, receipt of dialysis during hospitalization, baseline comorbidity of cancer, AKI at admission, baseline lymphocyte count, baseline potassium, and low-density lipoprotein cholesterol levels. The predicted AKI non-recovery risk model using the eXtreme Gradient Boosting (XGBoost) algorithm achieved an area under the receiver operating characteristic (AUROC) curve statistic of 0.807, discrimination with a sensitivity of 0.724, and a specificity of 0.738 in the temporal validation cohort.

**Conclusion:**

The machine learning model approach can accurately predict AKI non-recovery using routinely collected health data in clinical practice. These results suggest that multifactorial risk factors are involved in AKI non-recovery, requiring patient-centered risk assessments and promotion of post-discharge AKI care to prevent AKI complications.

## Introduction

Acute kidney injury (AKI) is associated with increased worsening long-term outcomes and direct medical costs (1–4). The recovery rate at hospital discharge or within 90 days from AKI ranges from 39.3 to 76.2% (5–7) and the recovery rate (free from dialysis therapy) of patients with AKI requiring dialysis at 90 and 365 days after dialysis initiation ranges from 47.7 to 56.6% (8). Timing of kidney function recovery after an AKI episode is associated with an increased risk of onset and long-term progression of chronic kidney disease and can affect survival rates (6–10). There is a need to improve the risk prediction for AKI non-recovery, and the predictive model may serve as a risk stratification tool to assist in the development of an appropriate post-AKI care plan at a time of hospital discharge.

Risk predictive models of AKI non-recovery using traditional statistical methods have achieved a C-statistic or area under the receiver operating characteristic curve (AUROC) of 0.61–0.76 in the validation models (11–13). The variance in AKI non-recovery predictive performance may be associated with heterogeneous AKI cohort, the definition of AKI recovery, and variable length of follow-up. A recent study suggested that clinical experts have selected features that can successfully predict AKI non-recovery within 7 days with an AUROC of 0.879 in the multi-center hospitalized cohort (14). These models tended to focus on patients with AKI undergoing dialysis or with critical illnesses. Machine learning approach using routinely collected health data in practice has demonstrated a promise for predicting AKI development in outpatient (15) or inpatient settings (16, 17). Currently, there is not a single validated machine learning model to identify the risk predictors of AKI non-recovery to establish supporting strategies for post-discharge AKI care. This study aimed to develop and validate a machine learning model for predicting AKI non-recovery at hospital discharge for the development of targeted interventions to prevent long-term adverse kidney outcomes.

## Methods

### Study Population

We used electronic health record data from the AKI Recovery Evaluation Study for a hospitalized adult cohort between January 1, 2010, and December 31, 2017, from the network of Chang Gung Memorial Hospitals in Taiwan. This study used the Chang Gung Research Database described in previous articles (18). Briefly, the adult hospitalized cohort from the AKI Recovery Evaluation Study included hospitalized patients who had an AKI episode at admission (index_AKI) and/or during the hospitalization (hospital-acquired AKI, HA_AKI) for analyzing the kidney function recovery after AKI exposure (as shown in Supplementary Material and Supplementary Table 1).

This study was approved by the Institutional Review Board of Chang Gung Memorial Foundation at Taipei, Taiwan, which approved a waiver of patient consent due to the retrospective nature of the study and use of de-identified data (permit number: 201901312B0C502). All datasets used in this study were de-identified prior to being transferred to the study investigators. The study followed the Transparent Reporting of a Multivariable Prediction Model for Individual Prognosis or Diagnosis (TRIPOD) statement and recommendations for reporting machine learning analyses in the clinical research for reporting predictive model development and validation (19, 20).

### Study Outcomes

The outcome of AKI non-recovery was defined as a serum creatinine (SCr) value obtained close to the date of discharge (during the index hospitalization or within 3 days following discharge) >1.5-fold of the baseline SCr value (within 3 months before AKI hospitalization) (21). Patients without SCr data during their hospital stay and within 3 days post-AKI discharge (it is common to have the first follow-up visit in outpatient setting) and who died during the hospitalization were excluded from the analysis. Details of pre-hospitalization baseline SCr value are presented in Supplementary Material/Definition of the AKI cohort section.

### Candidate Features and Imputation of Missing Data

Primarily, 64 candidate predictor features were available prior to and during the index hospitalization for AKI based on a review of the literature and clinical experience (as shown in the Supplementary Material/Candidate features section). These predictor features were patient demographic and clinical characteristics, laboratory results, nephrotoxic medication, and healthcare services utilization in pre-hospitalization and during the hospitalization. To increase the generalizability of the predictive model for both critical and non-critical ill hospitalized patients, absence of laboratory results was determined by the groups of patients with and without the use of intensive care units (ICUs) services to fill in the missing feature's values with the medians of the corresponding feature in the group to obtain the best model performance and maintain both sensitivity and specificity above 70% (Supplementary Material/Missing data imputation section/Supplementary Tables 1, 3).

### Feature Selection

The recursive feature elimination based on the random forest was employed to determine the number of features that should be included during model training from all candidate features (*n* = 63) by constructing the same model repeatedly using caret package in R and random forest as a basic model. The result indicated that the model with 20 features can achieve the best performance (Supplementary Figure 1). We used XGBoost to train the model and determine the 20 most important features (Table 1). Details of feature selection are presented in the Supplementary Material/Feature selection. This was to reduce the model complexity and prevent the final predictive model from overfitting that enables clinical applications. The distribution of each variable between recovery and non-recovery AKI groups was compared with chi-square test for categorical data and independent *t*-test was for means of continuous data.

**Table 1:** Top 20 important features for predicting acute kidney injury (AKI) non-recovery.

**Selected features**	**Derivation cohort**				**Temporal validation cohort**			
	***N* (*n* = 8,600)**	**Recovery (*n* = 4,729)**	**Non-Recovery (*n* = 3,871)**	***P* value**	***N* (*n* = 2,866)**	**Recovery (*n* = 1,580)**	**Non-recovery (*n* = 1,286)**	***P* value**
Age at index date, mean (SD)	8,600	66.53 (15.05)	64.39 (16.09)	<0.0001	2,866	67.08 (15.35)	64.20 (15.92)	<0.0001
HA-AKI, *n* (%)	1,257	356 (7.53)	901 (23.28)	<0.0001	406	124 (7.85)	282 (21.93)	<0.0001
Index AKI stage at index admission, *n* (%)				<0.0001				<0.0001
Stage 2	1,384	586 (12.39)	798 (20.61)	524	217 (13.73)	307 (23.87)		
Stage 3	3,961	2,595 (54.87)	1,366 (35.29)	1,205	780 (49.37)	425 (33.05)		
Chalrson comorbidity index (<1 year before index admission), *n* (%)								
Chronic kidney disease	2,774	2,062 (43.60)	712 (18.39)	<0.0001	893	648 (41.01)	245 (19.05)	<0.0001
Cancer	2,332	951 (20.11)	1,381 (35.68)	<0.0001	784	331 (20.95)	453 (35.23)	<0.0001
Baseline laboratory results (< = 7 days before index admission), mean (SD)								
Index_SCr, mg/dL	8,600	4.65 (3.19)	2.96 (2.25)	<0.0001	2,866	4.23 (3.07)	2.89 (2.26)	<0.0001
Baseline_SCr, mg/dL	8,600	3.29 (2.91)	1.30 (1.13)	<0.0001	2,866	2.91 (2.69)	1.26 (1.13)	<0.0001
Blood urea nitrogen (BUN), mg/dL	5,965	46.15 (30.19)	26.58 (20.69)	<0.0001	1,938	43.17 (30.39)	26.14 (20.80)	<0.0001
Potassium (K), mEq/L	6,559	4.25 (0.82)	4.07 (0.72)	<0.0001	2,081	4.26 (0.80)	4.00 (0.67)	<0.0001
Low density lipoprotein cholesterol (LDL), mg/dL	2,820	98.11 (31.86)	100.70 (31.20)	0.0351	1,080	99.06 (31.63)	96.00 (31.35)	0.1215
Serum uric acid (SUA), mg/dL	3,444	7.49 (2.30)	6.82 (2.41)	<0.0001	1,183	6.87 (2.33)	6.49 (2.52)	0.0093
Calcium (Ca), mg/dL	4,260	8.68 (0.77)	8.54 (0.70)	<0.0001	1,362	8.63 (0.74)	8.55 (0.76)	0.0548
C-reactive protein (CRP), mg/L	5,161	82.13 (81.26)	83.47 (78.11)	0.5495	1,840	79.99 (80.73)	84.96 (79.61)	0.1907
Albumin, g/dL	5,283	3.16 (0.65)	2.93 (0.67)	<0.0001	1,665	3.22 (0.65)	2.96 (0.66)	<0.0001
Erythrocyte sedimentation rate (ESR), mm/hr	305	50.11 (30.20)	42.06 (31.18)	0.0249	78	50.08 (33.40)	41.28 (31.09)	0.2317
White blood cell (WBC)x10^9^/L	7,760	9.30 (4.75)	8.98 (5.11)	0.0042	2,594	9.36 (4.81)	9.31 (5.21)	0.7851
Lymphocyte count (LPC), %	7,573	14.78 (9.69)	15.32 (11.59)	0.0297	2,565	14.48 (9.71)	15.38 (11.29)	0.0339
Neutrophil count (NPC),%	7,557	74.95 (12.67)	73.18 (14.88)	<0.0001	2,552	76.02 (12.24)	73.97 (14.20)	0.0001
Use of health service								
Number of outpatient visits < = 3 months before index date, mean (SD)	7,723	5.79 (6.54)	5.06 (4.16)	<0.0001	2,607	4.90 (4.63)	5.03 (4.08)	0.4551
Dialysis during the hospitalization, *n* (%)	1,718	993 (21.00)	1397 (36.09)	<0.0001	453	276 (7.47)	177 (13.76)	<0.0001

*p: chi-square test was performed for categorical data and independent t-test was for means of continuous data between recovery and non-recovery AKI groups*.

**Figure 1:**
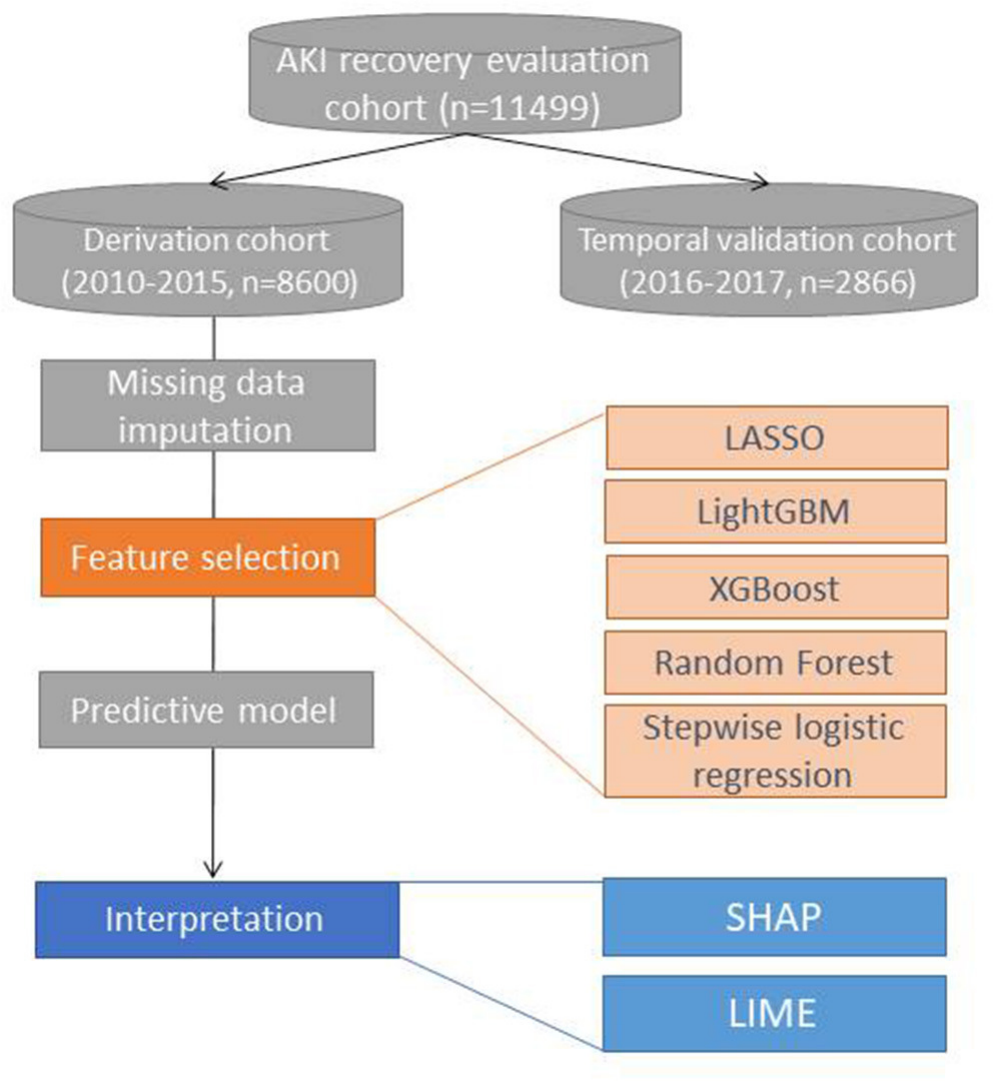
Study analysis flow. LR, logistic regression; LASSO, least absolute shrinkage and selection operator; XGBoost, eXtreme Gradient Boosting; LightGBM, Light Gradient Boosting Machine.

The selected 20 features were further trained in stepwise logistic regression and four machine learning classification algorithms, such as adaptive least absolute shrinkage and selection operator (LASSO), random forest, eXtreme Gradient Boosting (XGBoost), and Light Gradient Boosting Machine (LightGBM) (Figure 1; Supplementary Table 4).

### Development and Validation of the Predictive Model

A five-fold cross-validation was used to confirm the combination of features and the final model for the outcome predictive model. The predictive performance of model discrimination was analyzed with the AUROC. The cut-off point was determined based on the best Youden index (22), to determine sensitivity and specificity in the final model. The patients hospitalized from 2016 to 2017 were considered as the temporal validation cohort to validate the predictive performance and generalization of the best final predictive models (Table 2).

**Table 2:** Summary of model comparisons for predicting AKI non-recovery in the derivation cohort.

	**Stepwise LR**	**LASSO**	**XGBoost**	**LightGBM**	**Random forest**
**Full model**					
AUROC	0.790 ± 0.014	0.788 ± 0.018	0.805 ± 0.019	0.801 ± 0.010	0.787 ± 0.015
Sensitivity	0.779 ± 0.065	0.654 ± 0.030	0.651 ± 0.082	0.667 ± 0.058	0.726 ± 0.072
Specificity	0.657 ± 0.061	0.779 ± 0.037	0.803 ± 0.099	0.783 ± 0.053	0.750 ± 0.081
Precision	0.652 ± 0.028	0.784 ± 0.028	0.811 ± 0.062	0.750 ± 0.009	0.784 ± 0.040
F1_score	0.708 ± 0.023	0.713 ± 0.023	0.717 ± 0.034	0.738 ± 0.013	0.751 ± 0.024
**Top-20 model**					
AUROC	0.787 ± 0.015	0.787 ± 0.016	0.808 ± 0.015	0.798 ± 0.005	0.787 ± 0.015
Sensitivity	0.654 ± 0.032	0.657 ± 0.044	0.661 ± 0.037	0.694 ± 0.037	0.672 ± 0.064
Specificity	0.779 ± 0.049	0.775 ± 0.062	0.796 ± 0.050	0.748 ± 0.029	0.791 ± 0.063
Precision	0.785 ± 0.035	0.784 ± 0.040	0.800 ± 0.031	0.790 ± 0.007	0.800 ± 0.035
F1_score	0.713 ± 0.015	0.713 ± 0.018	0.723 ± 0.016	0.705 ± 0.016	0.728 ± 0.027

*LR, logistic regression; LASSO, least absolute shrinkage and selection operator; XGBoost, eXtreme Gradient Boosting; LightGBM, Light Gradient Boosting Machine; and AUROC, area under the receiver operating characteristic curve*.

*XGBoost with top-20 feature predictive results in the temporal validation cohort yielded an AUROC of 0.807, a sensitivity of 0.724, and a specificity of 0.738 (with the cut-off point = 0.471)*.

SHapley Additive exPlanations (SHAP) values, feature importance, summary plot, and dependency plot were used to explain the final predictive model (23). The Local Interpretable Model-Agnostic Explanations (LIME) method with four scenarios (true positive, false positive, true negative, and false negative outcome prediction) were randomly applied to elaborate the feature importance ranking and marginal effect in individual patient prediction (as shown in Supplementary Material/Interpretation of the predictive model) (24). All experimental analyses were performed using R (version 4.1.2; R Foundation for Statistical Computing, Vienna, Austria) and Python software (version 3.7.3; Python Software Foundation, Wilmington, DE, USA). The details of package, function, and parameters used in this study are presented in the Supplementary Material/Derivation and validation of the predictive model section.

## Results

### Characteristics of the Study Cohort

A total of 11,466 patients with AKI who survived at the index hospital discharge were included in the derivation and temporal validation cohorts (8,600 in the 2010–2015 derivation cohort and 2,866 in the 2016–2017 validation cohort) (Supplementary Figure 1). All 63 candidate features are reported in Supplementary Table 2. The mean age of the study cohort ranged from 64 to 67 years in both the derivation and validation cohorts, and male patients (>50%) were predominantly observed in the cohorts. The mean baseline (pre-hospitalization) SCr value was 1.3 (±1.13) mg/dl in patients with AKI non-recovery in both the derivation and validation cohorts. Moreover, the baseline SCr values were 2.91 (±2.69) and 3.29 (±2.91) mg/dl in patients with AKI recovery in the derivation and validation cohorts, respectively. The rates of dialysis and ICU utilization during hospitalization were 19.98% and 15.8%, 30% and 27.7% in the derivation and validation cohorts, respectively (Supplementary Table 1).

### Predicting AKI Non-recovery and Machine Learning Algorithm Comparisons

A similar proportion of AKI non-recovery (45%) at hospital discharge was observed in both the derivation and validation cohorts. Moreover, the 20 features identified (Table 1) by XGBoost to predict AKI non-recovery outcome had a higher AUROC (0.808, SD 0.015) in the five-fold cross-validation in the derivation models. This indicates that the selected 20 most important features to train the model can achieve almost the same performance as that using all variables (Supplementary Figure 1; Table 2).

In terms of prediction performance in the derivation models with top 20 features (Table 2; Supplementary Table 3), the AUROC values of each algorithm were closed among stepwise logistic regression (0.787 ± 0.015), least absolute shrinkage and selection operator (LASSO) (0.787 ± 0.016), random forest (0.787 ± 0.015), LightGBM (0.798 ± 0.005), and XGBoost models (0.808 ± 0.015). The model with the best prediction performance was the XGBoost model, with maximized sensitivity and specificity of 0.661 (±0.037) and 0.796 (±0.05), respectively (Table 2). When F1 score was examined, the random forest (0.728 ± 0.027) had a slightly higher F1 score than XGBoost model (0.723 ± 0.164) in Table 2. Based on AUROC, the XGBoost model was chosen to validate its predictive performance in the temporal validation cohort. The optimal cutoff value was 0.471 to achieve good discriminatory power for AKI non-recovery vs. AKI recovery outcomes with the AUROC, sensitivity, and specificity of 0.807, 0.724, and 0.738 in the validation cohort, respectively. In addition, precision value and F1 score of the XGBoost model in the validation cohort were 0.692 and 0.708, respectively.

### Interpretation of the Predictive Model

**Figure 2:**
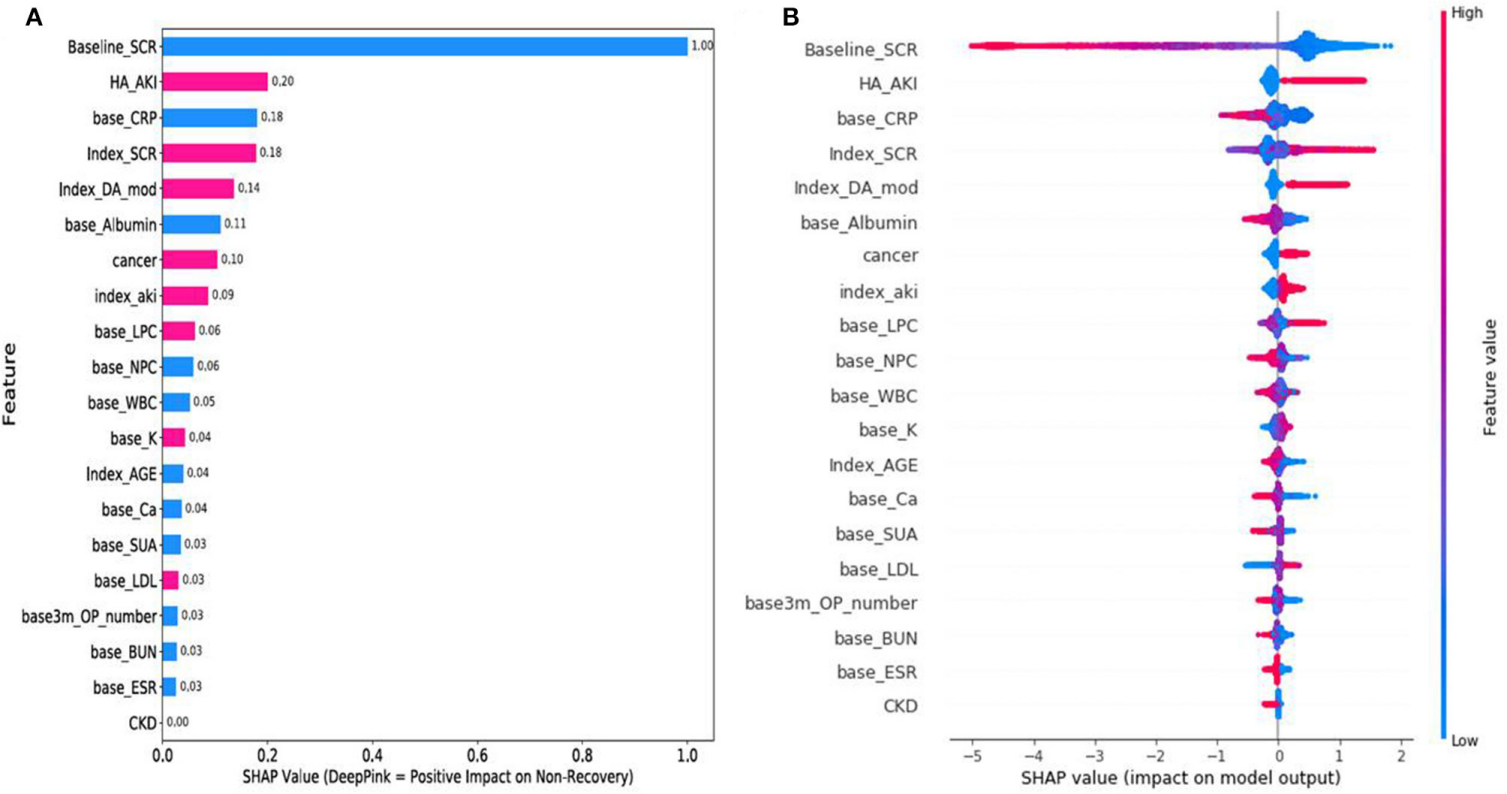
SHapley Additive exPlanations (SHAP) results. **(A)** SHAP feature importance. Eight features with deep pink color had a positive effect on AKI non-recovery prediction, 11 features with blue color were negatively correlated with AKI non-recovery prediction (1 with neutral), and a higher mean SHAP value has a higher effect on outcome prediction. **(B)** SHAP summary plot. The *x*-axis denotes the SHAP value for each feature, whereas the color represents the feature value (*y*-axis) from high to low (deep pink to blue). SHAP, SHapley Additive exPlanations and AKI, acute kidney injury.

The mean absolute SHAP value indicates individual feature importance in the XGBoost model (Figure 2A). Overall, eight of the top 20 characteristics, such as AKI occurring during hospitalization (HA_AKI), SCr level at admission (index_SCr), receipt of AKI dialysis during hospitalization (index_DA_mod), baseline comorbidity of cancer, AKI at admission (index_AKI), baseline lymphocyte count (LPC%), and baseline potassium (K, mEq/L) and low-density lipoprotein cholesterol (mg/dl) levels, were sorted by decreasing importance in predicting AKI non-recovery outcomes (with deep pink color in Figure 2A). The summary plot (Figure 2B) shows that a low baseline SCr level increases the importance of non-recovery prediction, whereas a high baseline SCr level can greatly reduce the importance. Furthermore, SHAP dependency plot (Supplementary Figure 2) demonstrates the interaction effect of two features on AKI recovery prediction.

The LIME visualization results of four random individual prediction scenarios are shown in Supplementary Figure 3. Supplementary Figures 3A–D provide an insight as to why the model makes a correct or incorrect prediction on an individual patient. Scenario A (ID 2231) and scenario B (ID2388) patients had similar characteristics. However, the scenario B patient had a higher index_SCr (1.99 mg/dl), baseline white blood cell (base_WBC) count (14 × 10^9^/L), baseline calcium (base_Ca) (> 8.5 mg/dl), and base_K (> 4.1 mEq/l) levels and a lower baseline C-reactive protein (base_CRP) level (30.76 mg/L) than the scenario A patient (SCr index = 0.98, WBC = 1.6 × 10^9^/l, base_Ca = 8.01 mg/dl, base_K = 3.1 mEq/l, and base_CRP = 166.72 mg/l). Feature importance ranking for the AKI recovery prediction is shown in Supplementary Figures 3C,D. High baseline SCr level (>3.54 mg/dl), without HA_AKI, and without prior cancer were the three most important features positivity correlated with AKI recovery prediction. Compared with the scenario C patient (a true AKI recovery prediction), the scenario D patient (a false AKI recovery prediction case) had a lower base_albumin (2.74 mg/dl), lower base_WBC count (4.8 × 10^9^/L), and lower SUA (5.1 mg/dl), but a higher calcium level (8.57 mg/dl) and higher number of nephrotoxic medication class used in outpatient setting (7 classes). These features could contribute false AKI recovery prediction in the scenario D.

## Discussion

The present study results demonstrate an explainable machine learning model for predicting kidney function non-recovery at hospital discharge in patients receiving inpatient AKI care from routinely collected health record data in clinical practice. Timing of AKI occurrence, receipt of dialysis, and SCr measurements; comorbidity of cancer; and pre-hospitalization baseline LPC% and K levels, which are relevant for predicting AKI non-recovery, did not return to a level ≥50% pre-hospitalization baseline SCr level. The SHAP and LIME methods enhance the interpretability of machine learning models and estimate the positive and negative contributions of each feature to the model prediction.

A previous study has identified four selected predictors (estimated glomerular filtration rate ≥ 30 ml/min/1.73 m^2^, preadmission hemoglobin level, platelet level, and diabetes comorbidity) of AKI recovery using stepwise logistic regression in patients with AKI undergoing dialysis therapy, however, failed to achieve acceptable discrimination performance (C-statistic, 0.645) 0.1 (3). Another study identified age, sex, SCr elevation, and urinary output as predictive factors for AKI recovery (without kidney replacement therapy within 5 days and SCr value <1.5 times the pre-ICU admission value) in patients requiring critical care, with an AUROC of 0.73 (65.4–80.8%) 0.1 (1). However, these studies have generally included patients requiring dialysis therapy in ICUs and have been limited to severe forms of AKI. To the best of our knowledge, this is the first study to develop an AKI recovery predictive model that uses machine learning algorithms for patients with AKI who were and were not admitted to ICUs. Importantly, this study included baseline SCr values, which are commonly ignored in previous studies assessing the risk factor for AKI recovery (25, 26). These previous studies have well-recognized limitations in the evaluation of AKI recovery. Based on the same data, our results showed that the XGBoost algorithm model achieved a good predictive performance with the 20 selected features and was reproducible in the validation cohort.

Another major contribution of this study includes its capability to derive reliable important features and validate an accurate AKI non-recovery predictive model based on a large AKI cohort with broad characteristic diversity. Given the poor outcomes among AKI survivors even in less severe AKI cases (3, 7), these study results of AKI non-recovery predictors can facilitate risk assessment for identifying at-risk patients for poor kidney recovery before hospital discharge and promote nephrology referrals for post-discharge care for patients who may benefit most from such interventions (27, 28).

Although the identified features should not be interpreted as etiologic factors or causal relationships, several temporal associations are noteworthy. These findings indicate that HA_AKI, high index SCr, and receipt of dialysis during the hospitalization were the three strongest predictors for non-recovery, a result consistent with those of prior studies (6, 10, 29). Because dialysis (intermittent and continuous hemodialysis) is a complementary therapy for AKI (30), it may be correlated with the severity of AKI. The fact that timing of AKI occurrence (AKI presence at admission [index_AKI] or during hospitalization [HA_AKI]) was also important to AKI non-recovery prediction, indicating that kidney function returned to the pre-hospitalization baseline level was not considered for hospital discharge in current practice. Further research is warranted to focus on long-term adverse outcomes among patients with AKI non-recovery who had a small improvement (e.g., recovery <50% baseline SCr) or worsening kidney function (e.g., increase > peak SCr) at hospital discharge.

The SHAP results showed that the baseline SCr value had bidirectional effects on AKI outcome prediction (Figure 2B). It is possible that patients with a high baseline SCr value (proxy of poor kidney function) or less severe episode of AKI at admission may contribute less to AKI non-recovery prediction (negative SHAP value in Figure 2B). In contrast, patients with a low baseline SCr value (blue color of feature value) may experience a severe episode of AKI at admission or during hospitalization, resulting in positive and significant effects on AKI non-recovery prediction (positive SHAP value in Figure 2B).

Cancer is an important factor for predicting AKI non-recovery. These results are consistent that cancer patients with AKI non-recovery within 7 days had a greater risk of end-stage kidney disease or death (14). We hypothesized that existing cancer influencing AKI non-recovery may reflect higher baseline risk of AKI progression because patients with cancer are at high risk for infections, sepsis, tumor lysis syndrome, and chemotherapy-associated toxicities (31, 32).

Although the relevance is low, the baseline lymphocyte count and low-density lipoprotein cholesterol level increased, positively predicting AKI non-recovery. Lymphocytes are associated with the systemic inflammatory response; the proportion of baseline LPC and its importance to AKI non-recovery prediction are not linear. Moreover, its relevance can be associated with hospital-acquired AKI (Supplementary Figure 2A). These laboratory results suggested that inflammatory response at baseline could mediate the recovery phase of AKI (33). The SHAP dependence plot (Supplementary Figure 2B) shows that baseline *K* value > 4 mEq/L increased the prediction of AKI non-recovery (Supplementary Figure 2B). In contrast, patients with *K* > 5 mEq/L and high SCr value at admission had a negative association with AKI non-recovery prediction, which could be due to further medical interventions during the inpatient stay.

Despite the increasing interest in the relative predictive performance of different machine learning approaches, the results of this study are significant as they contribute to a better understanding of the effect (positive or negative correlation) of each feature of the predictive AKI non-recovery outcomes and a possible explanation of model's misclassification in the SHAP and LIME procedures. For instance, the LIME results potentially provided an in-depth explanation for why the scenario B patient (ID 2388), having a similar baseline SCr level with the scenario A patient (ID 2231 with < 1.0 mg/dl), was misclassified as AKI non-recovery (the scenario B patient recovered) in the final predictive model (Supplementary Figures 3A,B). All SHAP and LIME results suggested that to further perform an external validation in different populations, a study investigating the association between time-varying feature values (e.g., SCr trajectory) and mechanisms of AKI non-recovery should be conducted in the future.

This study has some limitations. First, only routinely available clinical data in the electronic health records were used to identify clinical phenotypes. Other data, such as urine output, fluid status, inpatient medication use, or pathogen variables during the hospitalization in the derivation model that could change classification, were not assessed. Additionally, considering the changes to the time window for AKI recovery, the measurable candidate feature inclusions may affect the selected features and outcome predictive performance. However, this does not undermine the current findings but rather highlights the need for further studies to better understand the complex mechanisms correlated with AKI non-recovery. Second, missing data were common for some laboratory results included in the derivation models. Although different approaches of data imputation were examined and the best calibration method was used to preserve at least 70% sensitivity and specificity, the imputed feature's value could bias classification. Finally, the characteristics of patient and clinical data were derived initially from a single integrated health system in Taiwan. Although feature distribution and frequency were found to be generalizable in the derivation and temporal validation cohorts, further external cohort validation is necessary, especially using data from different healthcare systems and more recent clinical data in academic center cohorts.

In this study, XGBoost demonstrated good discrimination performance for AKI non-recovery at hospital discharge. In the temporal validated model, 20 important patients and clinical characteristics reflect the complexity of factors affecting the non-recovery kidney function. The eight characteristics supporting AKI non-recovery prediction should be considered risk predictors of adverse AKI outcome in further risk profiling to drive the development of post-AKI care strategy for inpatients with AKI before hospital discharge.

## Data Availability Statement

The original contributions presented in the study are included in the article/Supplementary Material, further inquiries can be directed to the corresponding author/s.

## Ethics Statement

This study was approved by the Institutional Review Board of Chang Gung Memorial Foundation at Taipei, Taiwan. Written informed consent for participation was not required for this study in accordance with the national legislation and the institutional requirements.

## Author Contributions

C-NH and Y-LT were involved in concept and design. C-NH and C-LL were involved in the drafting of the manuscript. C-LL and Y-CL performed statistical analysis. C-NH obtained funding. All the authors were involved in critical revision of the manuscript for important intellectual content.

## Funding

This study was funded by Kaohsiung Chang Gung Memorial Hospital (CMRPG8K0101 and CORPG8L0041 to C-NH). The funders had no role in the design and conduct of the study; collection, management, analysis, and interpretation of the data; preparation, review, or approval of the manuscript; and decision to submit the manuscript for publication.

## Conflict of Interest

The authors declare that the research was conducted in the absence of any commercial or financial relationships that could be construed as a potential conflict of interest.

## Publisher's Note

All claims expressed in this article are solely those of the authors and do not necessarily represent those of their affiliated organizations, or those of the publisher, the editors and the reviewers. Any product that may be evaluated in this article, or claim that may be made by its manufacturer, is not guaranteed or endorsed by the publisher.
