# Supplementary material for: Prediction and Clinically Important Factors of Acute Kidney Injury Non-recovery
Source: Front Med (Lausanne). 2022 Jan 17;8:789874. doi: 10.3389/fmed.2021.789874 (PMC8801583; doi:10.3389/fmed.2021.789874)
Supplement: Supplementary file 1 [file Data_Sheet_1.pdf]

Prediction and clinically important factors of acute kidney injury nonrecovery

Supplementary Material

Supplementary Methods

Supplementary References

Supplementary Table S1. KDIGO acute kidney injury staging definition

Supplementary Table S2. Candidate features for predicting acute kidney injury nonrecovery at hospital discharge

Supplementary Table S3. Comparison of the top-20 features models for predicting acute kidney injury nonrecovery

Supplementary Figure S1. Number of predictor selection and outcome of cross-validation

Supplementary Figure S2. SHapley Additive exPlanations dependence plot for lymphocyte count % and hospital-acquired acute kidney injury

Supplementary Figure S2. Local Interpretable Model-Agnostic Explanations for discriminating true and false predictions

Supplementary Methods

Acute Kidney Injury Recovery Evaluation Study cohort

This study comprised hospitalized patients aged 20 years or older between January 1, 2010 and December 31, 2017 from the network of Chang Gung Memorial Hospitals (CGMHs), including two medical centers, two regional hospitals, and three district hospitals located in different cities from north to south Taiwan. To assess kidney function recovery following acute kidney injury (AKI) exposure, hospitalized patients who had at least one serum creatinine (SCr) value both at admission and ≤ 90 days prior to the index hospitalization and survived at index hospital discharge were included in this retrospective cohort of Acute Kidney Injury Recovery Evaluation Study (AKIRES).

Patients who did not have SCr values both at admission and ≤ 90 days prior to the index hospitalization were first excluded. Patients undergoing kidney transplant or receiving maintenance dialysis therapy before the index hospitalization and aged younger than 20 years at the index admission date were also excluded. Patients with no SCr value during the hospital stay and within 3 days post-AKI discharge and who died during the hospitalization were excluded from the analysis. AKI recovery was defined as the last SCr value close to date of discharge during the index hospitalization or within 3 days following the discharge < 1.5-fold baseline SCr value (within 3 months before the AKI hospitalization).

Data sources

AKIRES data were obtained from the Chang Gung Research Database (CGRD), a de-identified, electronic health record database of patient information from the healthcare delivery system in Taiwan. The CGRD contains International Classification of Diseases, Ninth/Tenth Revision, Clinical Modification codes, Healthcare Common Procedure Coding System codes, Anatomical Therapeutic Chemical Classification System codes, and laboratory test results in emergency departments and in- and out-patient settings. The CGMHs (9584 beds) have accounted for approximately 11% of

Taiwan's national health insurance (NHI) program's annually covered health services in 2018, including over 9.1 million emergency and outpatient department visits and 300 000 hospital admissions.[S1] The Taiwan NHI program covers greater than 99% of the 23 million population and includes comprehensive health services since 1997.[S2] The study was approved by the Institutional Review and Ethics Board of CGMH, Taipei, Taiwan (permit number: 201901312B0C502).

Definition of the acute kidney injury cohort

Patients who had AKI at the index hospital admission (index_AKI) were defined with community-acquired AKI according to the modified Kidney Disease: Improving Global Outcomes criteria (Supplementary Table S1) as **SCr level increases within 48 hours with an increase of 0.3 mg/dL from baseline value and increases of ≥ 1.5 of baseline value (or increase to 4 mg/dL) within 7 days or up to 90 days prior to patient's first admission (index date) in the study period.[S3, S4] Baseline SCr level was first retrieved based on the availability of measured SCr within 2 days prior to the index date, then within 7 days for patients without a recent 2-day SCr, and then within 30 days or up to 90 days for those patients without any SCr ≤ 7 days.**

Two approaches were used to determine baseline SCr level. First, for patients who had multiple SCr levels in the 2- and 7-day time windows, the latest SCr (i.e., close to the index date) was selected as the baseline SCr level. Second, the mean SCr value within 8–90 days before the index date was defined as the baseline SCr value for patients who had multiple SCr measurements in the period.[S4,S5]

Hospital-acquired AKI was based on the highest SCr value during the index hospitalization compared with the index SCr value at admission as the peak SCr value was > 1.5 × SCr value at admission. Discharge SCr value was ascertained close to or within 3 days after the hospital discharge date.

Candidate features

Pre-hospitalization

Based on a literature review of factors shown to increase the risk of AKI hospitalization and expert opinions,[S6,S7] the features of interests are as follows:

Patient characteristics: age at the index hospital admission, sex, and 17 individual conditions of Charlson Comorbidity Index[S8] within 1 year before admission

Use of healthcare services: number of outpatient visits, emergency department visits, hospitalizations, and dialysis treatments in the preceding 3 months before index admission was ascertained

Use of potential nephrotoxic medication: Sixteen pharmacological classes were identified by Anatomical Therapeutic Chemical (ATC) Classification System codes in outpatient setting within 3 months prior to the index date: (1) analgesics (non-steroidal anti-inflammatory drugs or cyclooxygenase-2 inhibitors), (2) analgesics (opioids), (3) antiepileptics (gabapentin or phenytoin), (4) antihypertension (renin-angiotensin system inhibitor and potassium [K]-sparing diuretics), (5) contrast media, (6) oral hyperglycemic agent (non-metformin and metformin-based), (7) immunosuppressants, (8) anti-hyperuricemics, (9) anti-inflammatory/intestine agents, (10) antihistamines/antipsychotics/antispasmodics, (11) bisphosphonates, (12) digoxin, (13) lipid-lowering agents (statins, fibrates), (14) nitrates, (15) anticoagulants, and (16) antimicrobial agents. Sum of antimicoribal agents categoy used (2 sum) and sum of class of nephrotoxic medication used in the outpatient setting (base3m_OP_number).

Laboratory results: Most recent data of 19 items of laboratory results were retrieved +/–7 days of the index date as shown in Supplementary Table S1.

During hospitalization

Use of intensive care units (ICUs) and a receipt of dialysis with any modality during the index hospitalization period were analyzed. Overall, 63 features were analyzed after the exclusion of one

feature with missing data with 99.7% of the study cohort (serum total protein) (Supplementary Methods Table 3 and Supplementary Table S1).

Missing data imputation

Three methods were employed to impute the missing values, and the method that would make the model perform better was selected (Supplementary Methods Table 1): (1) stratifying the features with missing values according to the patient's index AKI severity (stages 1, 2, and 3) and filling the missing values with the median of the corresponding feature in each stratum, (2) stratifying the features with missing values according to the use of ICU and then filling the missing values with the median of the corresponding feature in the group and (3) using the *k* nearest neighbor data points to estimate the missing value, calculating the Euclidean distance based on each missing data point to determine the *k* nearest data points, and calculating the distance-weighted average of the data to fill.[S9] For instance, the missing values of baseline blood urea nitrogen, K, and phosphorus were filled with the median value in the ICU and non-ICU groups (Supplementary Methods Table 2). Supplementary Methods Table 3 presents the rate of missing of individual laboratory result.

Supplementary Methods Table 1. Missing data imputation methods and results

Method	AUC	Sensitivity	Specificity
AKI stage	0.805±0.012	0.770±0.049	0.682±0.037
ICU	0.807 ±0.004	0.747±0.058	0.712±0.055
KNN algorithm	0.801±0.013	0.764±0.049	0.684±0.043

Supplementary Methods Table 2. Example of filling the median value of the variable

Group	Calculating the median of variables from each group		
ICU	base_BUN	base_K	base_P
0 (no)	29.4	4.1	4.25
1 (yes)	24	4	3.95

BUN: blood urine nitrogen; K: potassium; P: phosphate

Supplementary Methods Table 3. Rates of missing laboratory results data

Laboratory result	Missing rate	Laboratory result	Missing rate	Laboratory result	Missing rate
base_WBC	0.083	base_HB	0.310	base_CRP	0.610
base_LPC	0.174	base_K	0.436	base_TG	0.671
base_NPC	0.176	base_BUN	0.480	base_SUA	0.680
base_NLR	0.178	base_Albumin	0.570	base_Ca	0.694
base_LDL	0.705	base_P	0.796	base_TCHOL	0.909
base_HbA1c	0.712	base_ESR	0.949	base_T_protein_B	0.997
base_GAC	0.816				

One laboratory variable was excluded from feature selection process (base_protein_B: baseline serum total protein).

Feature selection

Recursive feature elimination (RFE) was employed to eliminate low-weight features by constructing the same model repeatedly using the "caret" package in R and random forest as a basic model with cross-validation to find the best number of features. The purpose is to select a feature subset that is most beneficial to the model performance. Feature selection is a NP-hard problem, meaning that it is an intractable problem when the number of features is enormous. In the present study, we used the RFE method based on random forest to determine the optimal number of features was 20 [S10], as shown in Supplementary Figure S1.

Supplementary Methods Table 4 presents the methods used to perform feature selection. Wrapper methods treat the selection of a feature subset as an optimization problem to generate different feature combinations, evaluate the combinations, and compare them with other combinations. It directly takes the model performance as the criterion for the evaluation of the feature subsets. In the embedded method, different from the wrapper methods, feature selection and the model training are two distinct processes, whereas the embedded feature selection is a combination of the feature selection and the model training. Moreover, both are in the same optimization process to complete,

that is, automatic feature selection during model training.

Supplementary Methods Table 4. Feature selection methods

Wrapper method	Embedded method
• Recursive feature elimination based on random forest (RFE)<br>• Stepwise logistic regression	• Least absolute shrinkage and selection operator<br>• eXtreme Gradient Boosting<br>• Light Gradient Boosting Machine

Derivation and validation of the predictive model

Once the aforementioned feature selection procedure is completed, we use the features selected by each method to build predictive models and use fivefold cross-validation in the derivation cohort (training dataset). Taking the performances of fivefold cross-validations as the criteria to confirm the combination of features and the model that will be used as the final predictive model, the performance of the models was measured in the area under the receiver operating characteristic curve. To verify the generalization and robustness of the predictive model, we used the testing dataset of patients admitted from 2016 to 2017 (temporal validation cohort). There is no data leakage problem in this standard procedure of development and validation of machine learning prediction. The software of packages, function and parameters used in this study are listed as following:

Step	Algorithm	Package/Method	Parameter
Number of feature selection from all candidates	Recursive Feature Elimination (RFE)	caret	
Top 20 important feature selection	XGBoost	caret/<br>xgbTree	nrounds = 50<br>max_depth = 3<br>eta = 0.3<br>gamma = 0<br>colsample_bytree = 0.6<br>min_child_weight = 1<br>subsample = 1
Model comparisons in derivation dataset (2010-2015 hospitalized cohort)	Logistic Regression	caret/<br>glm	
	Lasso	caret/<br>glmnet	tuneGrid = expand.grid(alpha = 1,lambda=seq(0.0001, 1, length = 20))
	XGBoost	caret/	nrounds = 50

		xgbTree	max_depth = 3<br>eta = 0.3<br>gamma = 0<br>colsample_bytree = 0.6<br>min_child_weight = 1<br>subsample = 1
	Random Forest (RF)	caret/<br>rf	preProc = c("center", "scale"),<br>family = "binomial"<br>metric="ROC"
	LightGBM (Python 3.8.3)	lightgbm	boosting_type: ['gbdt']<br>'colsample_bytree': [1.0],<br>'importance_type': ['gain'],<br>'learning_rate': [0.01],<br>'max_depth': [6],<br>'min_child_samples': [21],<br>'min_child_weight': [0.01],<br>'min_split_gain': [0.0],<br>'n_estimators': [9620],<br>'num_leaves': [40],<br>'reg_alpha': [0.633],<br>'reg_lambda': [0.633],<br>'silent': [True],<br>'feature_fraction': [0.7],<br>'bagging_fraction': [1],
Temporal validation (2016-2017 hospitalized cohort)	XGBoost	caret/<br>xgbTree	nrounds = 600<br>max_depth = 6<br>eta = 0.015<br>gamma = 0.7<br>colsample_bytree = 0.6<br>min_child_weight = 3<br>subsample = 0.5

Interpretation of the predictive model

The SHapley Additive exPlanations (SHAP) technique assigns an importance value to each feature of interest for a specific outcome prediction.[S11] Using bootstrapped SHAP values, we can observe the AKI nonrecovery predictive model by evaluating the marginal effects of predictive features identified by the model. SHAP is a game theoretical approach used to explain the outputs of the machine learning models. The intent of the explainer in this study is to explain the results predicted by the model and to identify the influential features. Each feature is assigned an importance value, SHAP value, which represents its effect on the model prediction. Thus, the SHAP value can be a unified measure of feature importance.

Figure 2A presents the global effect of each feature on the outcome prediction. Top-100 is an arbitrary cutoff used to analyze the most important features to illustrate heterogeneity. Notably, a higher average SHAP value indicates a higher effect on AKI nonrecovery outcome prediction. Similar with the coefficients in the linear regression, SHAP values can have positive and negative values to present the positive and negative contributions of each feature to the model prediction. The SHAP summary plot shows whether a feature has a negative or positive effect on outcome prediction (Figure 2B) and further brings the whole data to a single plot, in which each point represents a SHAP value for a feature of a data instance. The x-axis denotes the SHAP values, whereas the color represents the feature value (y-axis) from high to low (deep pink to blue). The features are ordered according to their importance, but it presents the distribution of the SHAP value for each feature (Figure 2B).

In addition, the SHAP dependence plot was used to present the effect of a single feature on model prediction when considering the interactive effect with another feature on the predictive outcome. A SHAP dependency plot (Supplementary Figure S2) is a scatterplot that presents the association between the values of a specific feature and their corresponding SHAP values.

Local Interpretable Model-Agnostic Explanations (LIME) can locally approximate a black-box model by a simple glass-box model in a faithful manner.[S12] Each individual prediction was characterized by a unique ranking profile of feature importance as the weight value (x-axis) and influence of each feature (y-axis) on outcome prediction. In the present study, we randomly sampled the validation dataset and used the LIME algorithm to fit the four different predictive behaviors of the model to the sample to verify the rationality of the basis of the model for predicting results. Supplementary Figure S3 presents a true AKI nonrecovery prediction case, false nonrecovery prediction case, true AKI recovery prediction case, and false AKI recovery case, respectively.

Supplementary References

1. National Health Insurance Administration. *2018 Annual Report of Health Services Claims, by Health Care Organizations*. <> (2020). Accessed Sept 3 2020.
2. Cheng SH, Chiang TL. The effect of universal health insurance on health care utilization in Taiwan: results from a natural experiment. *JAMA*. 1997;278(2):89-93.
3. Khwaja A. KDIGO clinical practice guidelines for acute kidney injury. Nephron Clin Pract. 2012;120(4):c179-84. PMID: 22890468. doi: 10.1159/000339789.
4. Thomas ME, Blaine C, Dawnay A, Devonald MA, Ftouh S, Laing C, et al. The definition of acute kidney injury and its use in practice. Kidney Int. 2015;87(1):62-73.
5. Hsu CN, Liu CL, Tain YL, Kuo CY, Lin YC. Machine learning model for risk prediction of community-acquired acute kidney injury hospitalization from electronic health records: development and validation study. *Journal of Medical Internet Research.* 2020;22:e16903.
6. Stucker F, Ponte B, De la Fuente V, Alves C, Rutschmann O, Carballo S, et al. Risk factors for community-acquired acute kidney injury in patients with and without chronic kidney injury and impact of its initial management on prognosis: a prospective observational study. BMC Nephrol. 2017;18(1):380.
7. Murugan R, Weissfeld L, Yende S, Singbartl K, Angus DC, Kellum JA. Association of statin use with risk and outcome of acute kidney injury in community-acquired pneumonia. Clin J Am Soc Nephrol. 2012;7(6):895-905.
8. Sundararajan V, Henderson T, Perry C, Muggivan A, Quan H, Ghali WA. New ICD-10 version of the Charlson comorbidity index predicted in-hospital mortality. J Clin Epidemiol. 2004 ;57(12):1288-94.
9. García-Laencina PJ, Sancho-Gómez J-L, Figueiras-Vidal AR, Verleysen M. K nearest neighbours with mutual information for simultaneous classification and missing data imputation. *Neurocomputing.* 2009;72(7-9):1483-1493
10. Wang Y, Tetko IV, Hall MA, et al. Gene selection from microarray data for cancer classification—a machine learning approach. *Computational biology and chemistry.* 2005;29(1):37-46.
11. Marco Tulio Ribeiro, Sameer Singh, and Carlos Guestrin. " Why should i trust you?" Explaining the predictions of any classifier". In: Proceedings of the 22nd ACM SIGKDD international conference on knowledge discovery and data mining. 2016: pp. 1135–1144.
12. Scott M Lundberg and Su-In Lee. "A unified approach to interpreting model predictions" In: Advances in neural information processing systems. 2017: pp. 4765–*4774.*

Supplementary Table S1. KDIGO acute kidney injury staging definition

AKI stage	Serum creatinine (SCr) change	Urine output
1	Increase in SCr of 0.3 mg/dL within 48 h or<br>an increase of 1.5- 1.9× the baseline value within the 7 days	<0.5 ml/kg/hour for 6-12 hours
2	Increase in Scr of 2.0–2.9× the baseline value	<0.5 ml/kg/hour for ≥12 hours
3	Increase in Scr of ≥3× the baseline value or a Scr of ≥4 mg/dL or initiation of kidney replacement therapy	<0.3 ml/kg/hour for ≥24 hours or anuria for ≥12 hours

Based on the AKI definition provided in the KDIGO clinical practice guideline[27]
KDIGO: Kidney Disease: Improving Global Outcomes; AKI: acute kidney injury

Supplementary Table S2 Candidate features for predicting acute kidney injury nonrecovery at hospital discharge

Candidate predictors	Derivation cohort				Temporal validation cohort			
	N (n=8,600)	Recovery (n=4,729)	Non-Recovery (n=3,871)	*P* value	N (n=2,866)	Recovery (n=1,580)	Non-Recovery (n=1,286)	*P* value
Age at index date, years, mean (SD)	8600	66.53 (15.05)	64.39 (16.09)	<.0001	2866	67.08 (15.35)	64.20 (15.92)	<.0001
**Basic characteristics, n (%)**								
Sex				0.6093				0.9105
Male	4807	2655 (56.14)	2152 (55.59)		1579	869 (55.00)	710 (55.21)	
Female	3793	2074 (43.86)	1719 (44.41)		1287	711 (45.00)	576 (44.79)	
HA-AKI**, n (%)**	1257	356 (7.53)	901 (23.28)	<.0001	406	124 (7.85)	282 (21.93)	<.0001
Index AKI stage at index admission**, n (%)**				<.0001				<.0001
Stage 1	3255	1548 (32.73)	1707 (44.10)		1137	583 (36.90)	554 (43.08)	
Stage 2	1384	586 (12.39)	798 (20.61)		524	217 (13.73)	307 (23.87)	
Stage 3	3961	2595 (54.87)	1366 (35.29)		1205	780 (49.37)	425 (33.05)	
**Charlson comorbidity index (<1 year before index admission), n (%)**								
Acute myocardial infarction	350	209 (4.42)	141 (3.64)	0.0696	78	40 (2.53)	38 (2.95)	0.4885
Congestive heart failure	1037	661 (13.98)	376 (9.71)	<.0001	302	176 (11.14)	126 (9.80)	0.2447
Peripheral vascular diseases	314	201 (4.25)	113 (2.92)	0.0011	63	35 (2.22)	28 (2.18)	0.9451
Cerebral vascular accident	1282	752 (15.90)	530 (13.69)	0.0042	365	223 (14.11)	142 (11.04)	0.0141
Dementia	356	222 (4.69)	134 (3.46)	0.0043	89	53 (3.35)	36 (2.80)	0.3942
Pulmonary disease	1006	547 (11.57)	459 (11.86)	0.6767	295	178 (11.27)	117 (9.10)	0.0575
Connective tissue disorder	129	71 (1.50)	58 (1.50)	0.9908	56	31 (1.96)	25 (1.94)	0.9724
Peptic ulcer	1468	818 (17.30)	650 (16.79)	0.5349	418	228 (14.43)	190 (14.77)	0.7952
Liver diseases	1770	830 (17.55)	940 (24.28)	<.0001	332	174 (11.01)	158 (12.29)	0.2894
Diabetes	3161	1954 (41.32)	1207 (31.18)	<.0001	965	571 (36.14)	394 (30.64)	0.0019
Diabetes complications	1088	751 (15.88)	337 (8.71)	<.0001	395	272 (17.22)	123 (9.56)	<.0001
Paraplegia	97	60 (1.27)	37 (0.96)	0.1716	395	272 (17.22)	123 (9.56)	<.0001
Renal disease	2774	2062 (43.60)	712 (18.39)	<.0001	893	648 (41.01)	245 (19.05)	<.0001

Cancer	2332	951 (20.11)	1381 (35.68)	<.0001	784	331 (20.95)	453 (35.23)	<.0001
Severe liver diseases	407	149 (3.15)	258 (6.66)	<.0001	96	34 (2.15)	62 (4.82)	0.0001
Metastatic cancer	692	225 (4.76)	467 (12.06)	<.0001	240	89 (5.63)	151 (11.74)	<.0001
HIV	4	4 (0.08)	0 (0.00)	0.0703	3	2 (0.13)	1 (0.08)	0.6877
**Nephrotoxic medicine use (<=3 months before index admission), n(%)**								
NSAIDs or COX II inhibitors (1a)	2277	1114 (23.56)	1163 (30.04)	<.0001	665	329 (20.82)	336 (26.13)	0.0008
Opioid analgesics (1b)	997	488 (10.32)	509 (13.15)	<.0001	260	115 (7.28)	145 (11.28)	0.0002
Gabapentin or Phenytoin (3)	70	40 (0.85)	30 (0.77)	0.7160	19	16 (1.01)	3 (0.23)	0.0106
Renin–angiotensin system inhibitors or potassium sparing diuretics (4)	3450	2047 (43.29)	1403 (36.24)	<.0001	1090	636 (40.25)	454 (35.30)	0.0066
Contrast media (5)	240	106 (2.24)	134 (3.46)	0.0006	85	37 (2.34)	48 (3.73)	0.0290
Non-metformin OHA (6a)	1894	1223 (25.86)	671 (17.33)	<.0001	594	373 (23.61)	221 (17.19)	<.0001
metformin OHA (6b)	589	325 (6.87)	264 (6.82)	0.9235	183	89 (5.63)	94 (7.31)	0.0679
Immunosuppressant (7a)	472	176 (3.72)	296 (7.65)	<.0001	182	92 (5.82)	90 (7.00)	0.1993
Uric acid lowering agent (8)	948	691 (14.61)	257 (6.64)	<.0001	345	241 (15.25)	104 (8.09)	<.0001
Anti-inflammation/intestine (9)	32	19 (0.40)	13 (0.34)	0.6173	11	6 (0.38)	5 (0.39)	0.9689
Antihistamines/ Antipsychotics/ Antispasmodics (10)	2197	1223 (25.86)	974 (25.16)	0.4588	719	398 (25.19)	321 (24.96)	0.8883
Bisphosphonates (11)	43	15 (0.32)	28 (0.72)	0.0079	8	4 (0.25)	4 (0.31)	0.7702
Digoxin (12)	221	126 (2.66)	95 (2.45)	0.5398	49	31 (1.96)	18 (1.40)	0.2481
Statins (13a)	1359	883 (18.67)	476 (12.30)	<.0001	479	300 (18.99)	179 (13.92)	0.0003
Fibrates (13b)	229	137 (2.90)	92 (2.38)	0.1359	68	46 (2.91)	22 (1.71)	0.0357
Nitrates (15)	993	656 (13.87)	337 (8.71)	<.0001	234	146 (9.24)	88 (6.84)	0.0197
Anti-coagulants (16)	736	353 (7.46)	383 (9.89)	0.0001	316	165 (10.44)	151 (11.74)	0.2695
Number of antimicrobials, mean (SD) (2sum)	8600	0.37 (0.64)	0.41 (0.67)	0.0240	2866	0.35 (0.65)	0.37 (0.64)	0.2850
Number of medication class, mean (SD) (3m_op_number)	7723	5.79 (6.54)	5.06 (4.16)	<.0001	2607	4.90 (4.63)	5.03 (4.08)	0.4551
**Baseline laboratory results, mean (SD)**								
Index_SCr, mg/dL	8600	4.65 (3.19)	2.96 (2.25)	<.0001	2866	4.23 (3.07)	2.89 (2.26)	<.0001

Baseline_SCr, mg/dL	8600	3.29 (2.91)	1.30 (1.13)	<.0001	2866	2.91 (2.69)	1.26 (1.13)	<.0001
Blood urea nitrogen (BUN), mg/dL	5965	46.15 (30.19)	26.58 (20.69)	<.0001	1938	43.17 (30.39)	26.14 (20.80)	<.0001
Potassium (K), mEq/L	6559	4.25 (0.82)	4.07 (0.72)	<.0001	2081	4.26 (0.80)	4.00 (0.67)	<.0001
HbA1c, %	3250	7.11 (1.79)	7.12 (1.74)	0.8506	1161	7.05 (1.67)	7.03 (1.68)	0.8684
Hemoglobin (Hb), mg/DL	6896	10.61 (1.98)	11.11 (2.02)	<.0001	2278	10.77 (2.03)	11.14 (2.06)	<.0001
Total cholesterol (TCHOL),mg/dL	883	172.00 (38.22)	173.59 (40.98)	0.5717	275	175.81 (40.16)	171.61 (43.62)	0.4078
Low density lipoprotein cholesterol (LDL), mg/dL	2820	98.11 (31.86)	100.70 (31.20)	0.0351	1080	99.06 (31.63)	96.00 (31.35)	0.1215
Triglyceride (TG), mg/dL	3274	141.58 (79.73)	140.36 (79.87)	0.6728	1087	143.05 (81.02)	130.87 (79.64)	0.0147
Serum uric acid (SUA), mg/dL	3444	7.49 (2.30)	6.82 (2.41)	<.0001	1183	6.87 (2.33)	6.49 (2.52)	0.0093
Calcium (Ca), mg/dL	4260	8.68 (0.77)	8.54 (0.70)	<.0001	1362	8.63 (0.74)	8.55 (0.76)	0.0548
Phosphorus (P),mg/dL	3278	4.52 (1.21)	3.97 (1.09)	<.0001	1031	4.49 (1.18)	3.94 (1.13)	<.0001
Albumin, mg/dL	5283	3.16 (0.65)	2.93 (0.67)	<.0001	1665	3.22 (0.65)	2.96 (0.66)	<.0001
Glucose AC, mg/dL	1049	139.13 (48.95)	137.07 (48.92)	0.5080	496	147.39 (47.88)	143.78 (51.98)	0.4326
C-reactive protein, mg/L	5161	82.13 (81.26)	83.47 (78.11)	0.5495	1840	79.99 (80.73)	84.96 (79.61)	0.1907
Erythrocyte sedimentation rate (ESR), mm/hr	305	50.11 (30.20)	42.06 (31.18)	0.0249	78	50.08 (33.40)	41.28 (31.09)	0.2317
White blood cell (WBC) x10^9/L	7760	9.30 (4.75)	8.98 (5.11)	0.0042	2594	9.36 (4.81)	9.31 (5.21)	0.7851
Lymphocyte count (LPC), %	7573	14.78 (9.69)	15.32 (11.59)	0.0297	2565	14.48 (9.71)	15.38 (11.29)	0.0339
Neutrophil count (NPC), %	7557	74.95 (12.67)	73.18 (14.88)	<.0001	2552	76.02 (12.24)	73.97 (14.20)	0.0001
**Use of health service**								
Prior dialysis, n (%)	1718	993 (21.00)	725 (18.73)	0.0088	453	276 (17.47)	177 (13.76)	0.0068
Number of outpatient visits<=3 months **before index date**, mean (SD)	7723	5.79 (6.54)	5.06 (4.16)	<.0001	2607	4.90 (4.63)	5.03 (4.08)	0.4551
Number of emergency department visits<=3months **before index date**, mean (SD)	4691	1.45 (0.91)	1.49 (1.06)	0.2223	1504	1.44 (0.92)	1.55 (1.07)	0.0359
Number of Hospitalization<= 3 months **before index date**, mean (SD)	2807	1.13 (0.38)	1.18 (0.47)	0.0032	932	1.12 (0.40)	1.22 (0.52)	0.0013

Intensive care unit during the hospitalization, n (%)	2587	1190 (25.16)	1397 (36.09)	<.0001	792	387 (24.49)	405 (31.49)	<.0001
Dialysis during the hospitalization, n (%)	1718	993 (21.00)	1397 (36.09)	<.0001	453	276 (7.47)	177 (13.76)	<.0001

HA-AKI: hospital-acquired acute kidney injury; NSAIDs: non-steroidal anti-inflammatory drugs; COX-2 inhibitors: cyclooxygenase-2 inhibitors

Supplementary Table S3 Comparison of the top-20 features models for predicting acute kidney injury nonrecovery

**Stepwise LR**		**LASSO**		**XGBoost**		**LightGBM**		**Random Forest**	
AUROC	0.787±0.015	AUROC	0.787±0.016	AUROC	0.808±0.015	AUROC	0.798±0.005	AUROC	0.787±0.015
Sensitivity	0.654±0.032	Sensitivity	0.657±0.044	Sensitivity	0.661±0.037	Sensitivity	0.694±0.037	Sensitivity	0.672±0.064
Specificity	0.779±0.049	Specificity	0.775±0.062	Specificity	0.796±0.050	Specificity	0.748±0.029	Specificity	0.791±0.063
Precision	0.785±0.035	Precision	0.784±0.040	Precision	0.800±0.031	Precision	0.790±0.007	Precision	0.800±0.035
F1_score	0.713±0.015	F1_score	0.713±0.018	F1_score	0.723±0.016	F1_score	0.705±0.016	F1_score	0.728±0.027
name	coefficient	name	coefficient	name	coefficient	name	coefficient	name	coefficient
Baseline_SCR	100	Baseline_SCR	100	Baseline_SCR	100	Baseline_SCR	100	Baseline_SCR	100
HA_AKI	53.97	Index_SCR	32.66	Index_SCR	29.72	Index_SCR	21.84	Index_SCR	51.7
Index_SCR	38.41	HA_AKI1	17.21	HA_AKI1	17.34	base_CRP	17.95	base_CRP	31.95
Index_DA	34.66	Index_DA	14.16	base_BUN	14.56	HA_AKI	14.31	base_WBC	29.67
Cancer	31.18	cancer	9.58	Index_DA	12.43	base_BUN	11.12	Index_AGE	29.24
base_CRP	27.07	base_CRP	8.45	base_CRP	12.39	base_Albumin	10.38	base_LPC	27.43
base_Albumin	24.11	base_Albumin	7.85	base_Albumin	9.37	Index_DA_mod	8.13	base_NPC	27.36
base_Ca	13.64	base_Ca	4.56	Cancer	7.52	base_WBC	7.22	base_BUN	27.22
Index_AGE	13.31	base_LPC	4.54	index_aki3	7.42	CKD	7.09	base_Albumin	24.7
base_LDL	11.92	Index_AGE	3.91	base_LPC	6.91	base_LPC	6.55	base_K	21.56
base_K	11.18	base_K	3.63	base_WBC	5.69	Index_AGE	5.74	base_Ca	17.28
base_WBC	11.05	base_WBC	3.52	base_Ca	4.55	base_NPC	5.05	base3m_OP_num	16.63
base_LPC	9.66	base_LDL	3.52	base_NPC	3.59	index_aki3	4.64	base_SUA	16.02
base_ESR	9.63	base_NPC	3.22	Index_AGE	2.95	base_LDL	3.57	base_LDL	16.02
base_NPC	6.78	index_aki3	3.1	base_SUA	2.8	base_SUA	3.55	HA_AKI1	11.2
index_aki3	5.6	base_ESR	2.98	CKD1	2.58	base_Ca	3.29	Index_DA	7.07
base_SUA	3.27	base_SUA	0.94	base_LDL	2.04	base_K	3.18	CKD1	4.91
base3m_OP_num	1.93	base3m_OP_num	0.58	base_K	1.9	cancer	3.17	cancer1	3.57
CKD	1.46	base_BUN	0.47	base3m_OP_num	0.93	base3m_OP_num	2.07	base_ESR	2.07
base_BUN	1.04	CKD	0.26	base_ESR	0.78	base_ESR	0	index_aki3	1.62

LR: logistic regression; LASSO: least absolute shrinkage and selection operator; XGBoost: eXtreme Gradient Boosting; LightGBM: Light Gradient Boosting Machine; AUROC: area under the receiver operating characteristic curve.

Supplementary Figure S1 Number of predictor selection and outcome of cross-validation

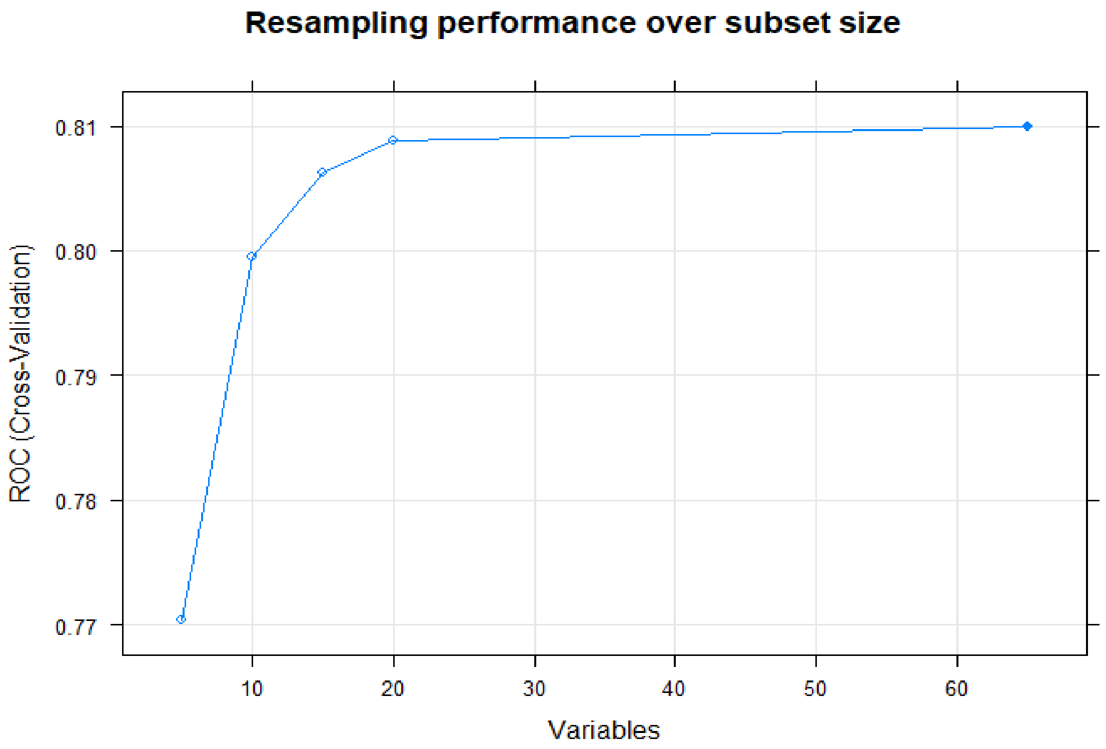

Number of variable=20 had a high AUROC of 0.808 (standard deviation 0.015) from the fivefold cross-validation in the derivation models.

Supplementary Figure S2 SHapley Additive exPlanations (SHAP) dependence plot for the target feature of base_LPC and base_K

A. When the value of the baseline lymphocyte count (base_LPC%) is <10% or >30%, the SHAP values increase rapidly, suggesting that these two conditions will affect renal recovery. Patients with hospital-acquired acute kidney injury (HA_AKI) have higher impact on nonrecovery prediction when their base_LPC% is <10%.

B. A low index_SCR measure increases the importance of AKI nonrecovery prediction when base_K is >4 mEq/dL. The color of the points corresponds to a second feature that may have an interaction effect with the target feature.

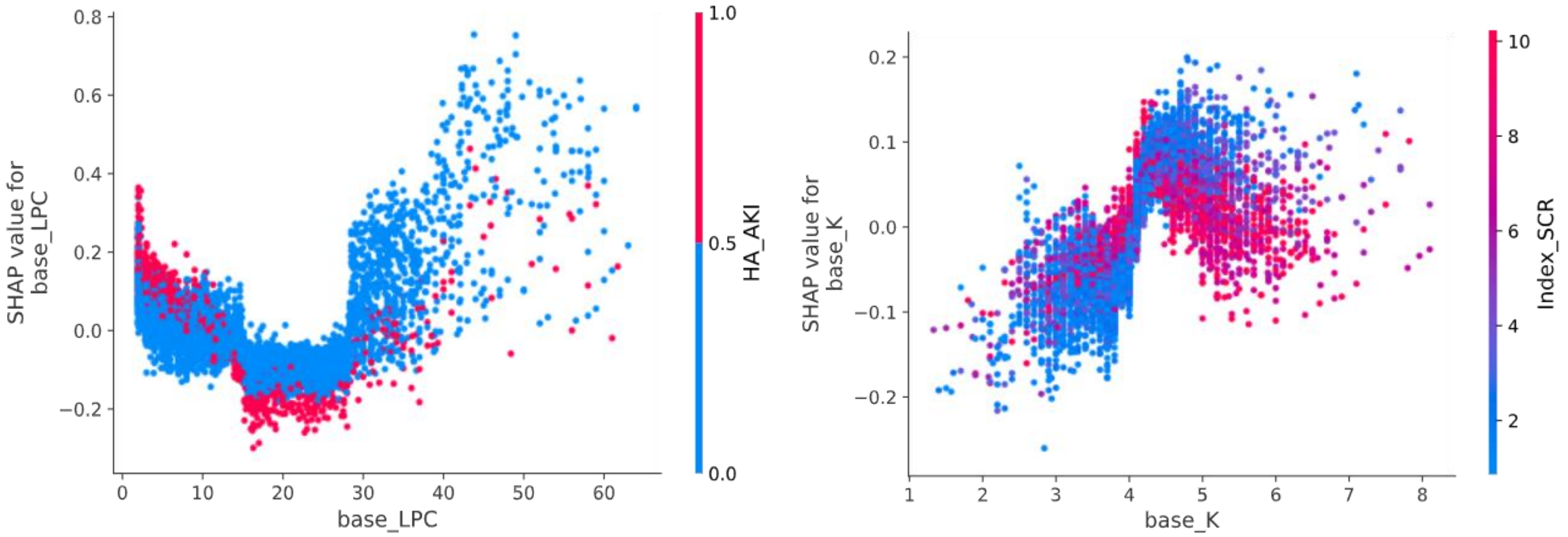

Supplementary Figure S3. Local Interpretable Model-Agnostic Explanations for discriminating true and false predictions

A. A patient with acute kidney injury (AKI) nonrecovery with a probability of 0.96; an accurate AKI nonrecovery prediction.

B. A recovery patient, predicted incorrectly by the model as nonrecovery with a probability of 0.948; a false AKI nonrecovery prediction.

C. A patient with AKI recovery with a probability of 0.992; an accurate AKI recovery prediction.

D. A patient with AKI nonrecovery, predicted incorrectly by the model as recovery with a probability of 0.942; a false AKI recovery prediction case

A: True AKI non-recovery

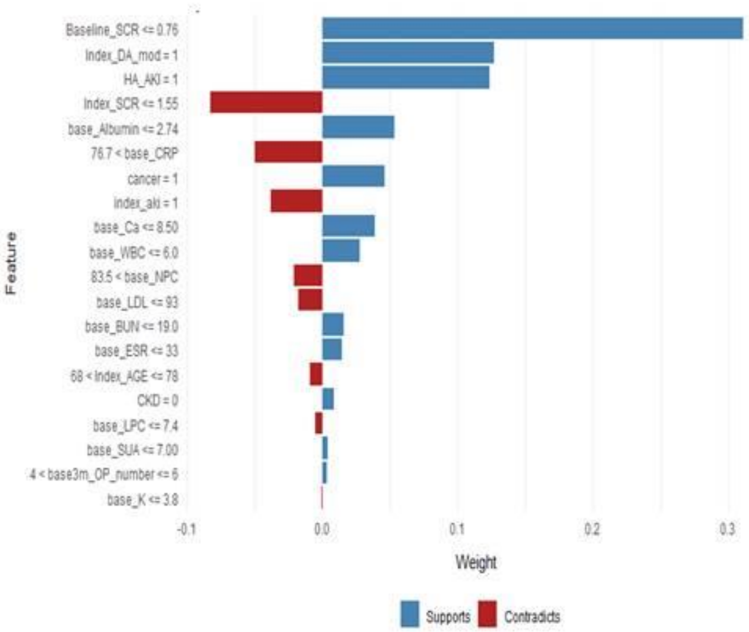

B: False AKI non-recovery

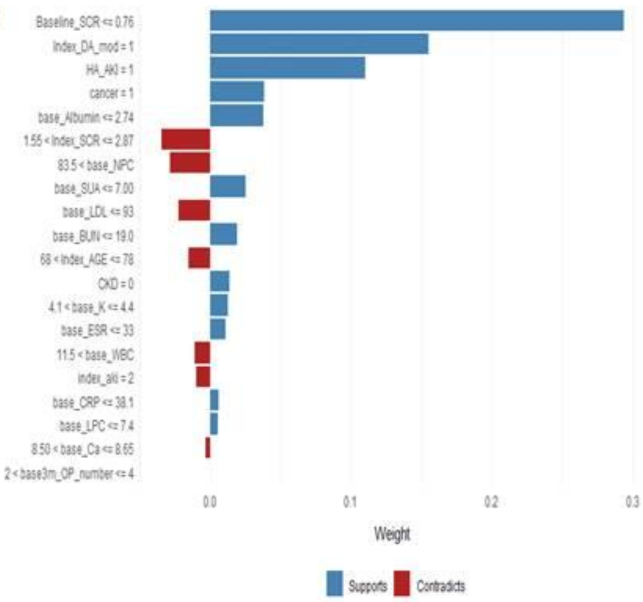

A: True AKI non-recovery		B: False AKI non-recovery	
**Predication probability**		**Predication probability**	
non-recovery (true)	0.960	non-recovery (true)	0.948
reovery (false)	0.040	recovery (false)	0.052
ID	2231	ID	2388
**Feature**	**Actual value**	**Feature**	**Actual value**
base_SCR	0.59	base_SCR	0.68
index_SCR	0.98	index_SCR	1.99
HA_AKI	yes	HA_AKI	Yes
base_BUN	13.3	base_BUN	10.1
index_DA_mod	Yes	index_DA_mod	Yes
base_CRP	166.72	base_CRP	30.76
base_Albumin	1.93	base_Albumin	2.01
base_LPC	2	base_LPC	7
Cancer	Yes	Cancer	Yes
index_aki	Stage 1	index_aki	Stage 2
base_WBC	1.6	base_WBC	14
CKD	No	CKD	No
index_AGE	70	index_AGE	76
base_NPC	85	base_NPC	88
base_Ca	8.01	base_Ca	8.62
base_SUA	7	base_SUA	4.62
base_K	3.1	base_K	4.3
base3m_OP_number	6	base3m_OP_number	3
base_LDL	93	base_LDL	93
base_ESR	33	base_ESR	33

C: True AKI recovery

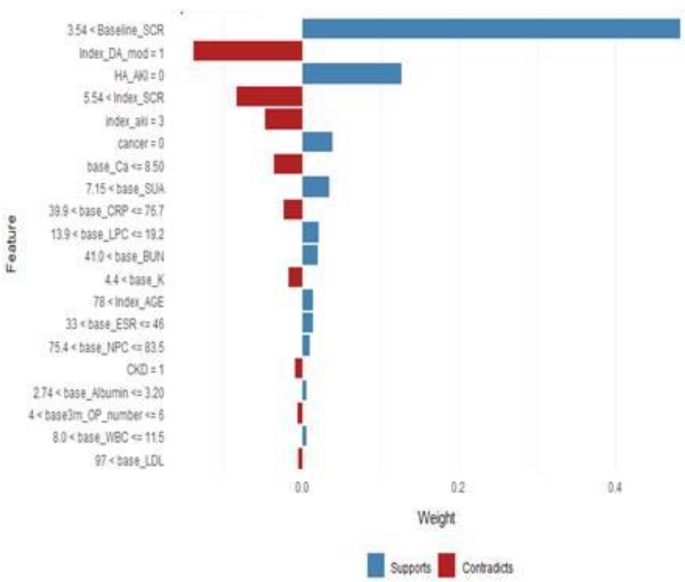

D: False AKI recovery

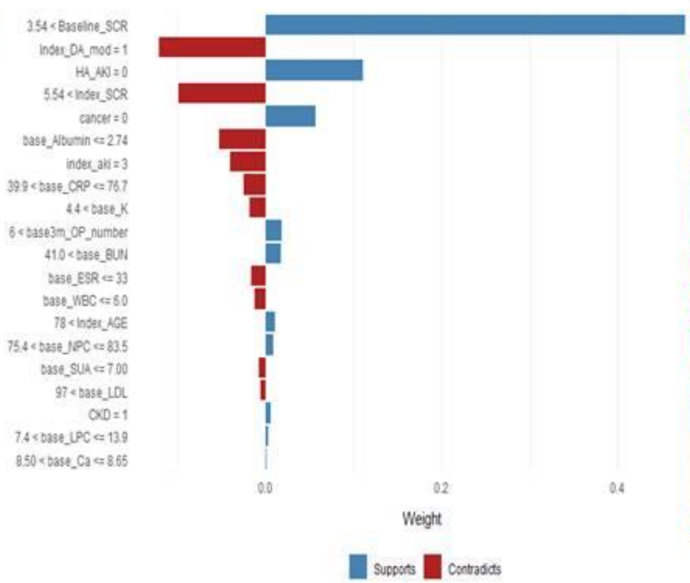

**C: True AKI recovery**		**D: False AKI recovery**	
**Predication probability**		**Predication probability**	
non-recovery (false)	0.008	non-recovery (false)	0.058
recovery (true)	0.992	recovery (true)	0.942
ID	1054	ID	2613
**Feature**	**Actual value**	**Feature**	**Actual value**
Baseline_SCR	8.32	Baseline_SCR	6.69
Index_SCR	10.42	Index_SCR	7.1
HA_AKI	No	HA_AKI	No
base_BUN	123.2	base_BUN	97
Index_DA_mod	Yes	Index_DA_mod	Yes
base_CRP	56.84	base_CRP	76.69
base_Albumin	3.19	base_Albumin	2.74
base_LPC	15	base_LPC	13.2
cancer	No	cancer	No
index_AKI	Stage 3	index_AKI	Stage 3
base_WBC	8.7K	base_WBC	4.8K
CKD	Yes	CKD	Yes
Index_AGE	90	Index_AGE	80
base_NPC	79	base_NPC	76.7
base_Ca	8.23	base_Ca	8.57
base_SUA	10.08	base_SUA	5.1
base_K	4.5	base_K	5.2
base3m_OP_number	5	base3m_OP_number	7
base_LDL	150	base_LDL	108
base_ESR	46	base_ESR	33
